# Correction: Research progress in architecture and application of RRAM with computing-in-memory

**DOI:** 10.1039/d3na90030d

**Published:** 2023-03-20

**Authors:** Chenyu Wang, Ge Shi, Fei Qiao, Rubin Lin, Shien Wu, Zenan Hu

**Affiliations:** a College of Mechanical and Electrical Engineering, China Jiliang University Hangzhou 310018 People's Republic of China shige@cjlu.edu.cn; b Dept of Electronic Engineering, Tsinghua University Beijing 100084 People's Republic of China

## Abstract

Correction for ‘Research progress in architecture and application of RRAM with computing-in-memory’ by Chenyu Wang *et al.*, *Nanoscale Adv.*, 2023, https://doi.org/10.1039/d3na00025g.

The authors regret that the author Ge Shi was not marked as the corresponding author and the affiliations were incomplete in the original manuscript. The corrected author list and affiliations are listed here.

The Royal Society of Chemistry apologises for these errors and any consequent inconvenience to authors and readers.

## Supplementary Material

